# Electroconvulsive Therapy: A Scotland-Wide Naturalistic Study of 4826 Treatment Episodes

**DOI:** 10.1016/j.bpsgos.2024.100434

**Published:** 2024-12-16

**Authors:** Julie Langan Martin, Rona J. Strawbridge, David Christmas, Michael Fleming, Stephen Kelly, Daphne Varveris, Daniel Martin

**Affiliations:** a School of Health and Wellbeing, University of Glasgow, Glasgow, United Kingdom; b Cardiovascular Medicine Unit, Department of Medicine Solna, Karolinska Institute, Stockholm, Sweden; c National Health Service Tayside, Ninewells Hospital, Dundee, United Kingdom; d National Health Service Greater Glasgow and Clyde, Glasgow, United Kingdom; e National Health Service Forth Valley, Stirling, United Kingdom

**Keywords:** Clinical Global Index-Severity Scale, Depression, Electroconvulsive therapy, Montgomery–Åsberg Depression Rating Scale, Response, Side effects

## Abstract

**Background:**

Electroconvulsive therapy (ECT) is an effective treatment option for several psychiatric disorders, including treatment-resistant depression, but there are concerns about potential adverse effects, particularly on cognition. This study describes ECT response and side effects in the Scottish ECT Audit Network.

**Methods:**

Data collected from 4826 treatment episodes includes pre-ECT and post-ECT illness severity scores (Clinical Global Impression-Severity [CGI-S] and Montgomery–Åsberg Depression Rating Scale [MADRS]), diagnosis, age, sex, consent status, treatment year, treatment frequency, dose, and reported side effects. Descriptive statistics were used to assess the response to ECT by diagnosis, and logistic regression was used to investigate which factors influenced ECT response and side-effect occurrence.

**Results:**

CGI-S scale scores were reduced after ECT in all diagnoses. For patients with depression or bipolar depression, MADRS scores were also reduced after ECT. The most common side effect was headaches (29%). Increased age and increased CGI-S scores were significantly associated (multiple-testing corrected *p* < .05) with better treatment response and more cognitive side effects.

**Conclusions:**

In a large observational outcome study of ECT, ECT appears to be effective (measured by reduction in CGI-S or MADRS scores) across a range of psychiatric diagnoses. Furthermore, increased age and increased illness severity scores at entry were the variables most significantly associated with treatment response and cognitive side effects.

## Plain Language Summary

We used the SEAN (Scottish Electroconvulsive Therapy [ECT] Audit Network) data to investigate whether ECT is effective. The SEAN was established in 1996 with the aim of improving ECT practice across Scotland to ensure safe, effective, patient-centered care. We used 11 years of data (∼4800 episodes of treatment) to explore the effectiveness of ECT and the frequency of side effects across 8 common mental illnesses. We also explored which individual or treatment factors contributed to the effectiveness of ECT and reported side effects.

We demonstrated that ECT was effective for depression, schizophrenia, schizoaffective disorder, bipolar disorder, mania, mixed affective disorders, personality disorder, and postpartum disorder. Headaches were reported in 29% of episodes during the treatment course, but other side effects such as manic switch and prolonged seizure were rare (<2%). Female sex and increased symptom severity were significantly associated with effectiveness and cognitive side effects.

Cognitive and memory problems may be reported in several psychiatric disorders such as severe depression and bipolar depression, with longer duration of illness being associated with more cognitive problems. Under the current guidelines, it is likely that the individuals included in this study will have tried multiple medications, over several years, before being offered ECT. Therefore, the side effects should be considered in the context of prolonged, severe, treatment-resistant illness.

Despite widely accepted efficacy in the treatment of a range of psychiatric disorders including major depressive disorder (1,2), bipolar disorder (3), schizophrenia (4,5), postpartum psychosis (6), and catatonia (7), electroconvulsive therapy (ECT) use continues to be questioned by some (8). Many researchers and clinicians have high regard for ECT because of its safety and efficacy (1), while others have concerns that it may negatively impact cognition and cerebral functioning (9).

Treatment recommendations for ECT are based on clinical assessments and broad clinical guidelines, including those from the National Institute for Health and Care Excellence (10). Despite these guidelines and the evidence of the effectiveness of ECT, there is significant variation in the use of ECT across geographical areas (11,12) and some evidence to suggest that the use of ECT varies by clinical team (13). Due to a combination of these factors, ECT is often used later than what could be considered optimal for patient outcomes (14).

In this study, we used the Scottish ECT Audit Network (SEAN) dataset, which is a large, naturalistic, clinical sample that includes all treatment episodes in adults across Scotland from 2009 to 2019. In this exploratory analysis, we aimed to investigate 1) whether ECT is effective for a range of psychiatric disorders (including depression, bipolar depression, schizophrenia, and mania), 2) which individual or treatment variables influence the response to ECT, and 3) which individual or treatment variables influence risk of side effects of ECT.

## Methods and Materials

### SEAN Dataset

From the SEAN, formerly the Scottish ECT Accreditation Network, information on all treatment episodes in adults across Scotland between 2009 and 2019 was obtained. SEAN was established in 1996 with the aim of improving ECT practice across Scotland to ensure safe and effective patient-centered care. It is one of the 11 Scottish National Audit Programmes delivered by Public Health Scotland. In June 2023, the Scottish ECT Accreditation Network changed to the SEAN to reflect a move toward a clinical audit framework. Each unit in Scotland where ECT is delivered collects information about ECT treatment delivery, patient outcomes, and adverse effects. This information is used to ensure safe practice and improve care and treatment. For most of the study period, 18 hospitals were delivering ECT across Scotland. While there may be local variations in practice, all ECT suites were part of the national audit and were subject to accreditation visits.

Outcomes measures collected by SEAN include pre- and post-treatment scores on the Montgomery–Åsberg Depression Rating Scale (MADRS), a widely used measure of depressive symptoms that is sensitive to change (15). Severity classification was defined as ≤10 = no depression (or remission), 10 to 30 = depression, and >30 = severe depression (16). The Clinical Global Impression-Severity (CGI-S) scale (17) was also used to obtain a global rating of severity measured on the following scale: 1 = normal, not ill at all; 2 = borderline mentally ill; 3 = mildly ill; 4 = moderately ill; 5 = markedly ill; 6 = severely ill; and 7 = extremely ill.

The information collected from medical records included year of treatment episode, age at treatment, sex, indication for ECT (i.e., primary ICD-10 diagnosis), number of treatments per episode, mean and maximum dose (millicoulombs, mC), total treatment dose (mC), maximum stimulations per treatment, anesthetic agents, electrode placement (bifrontal, bilateral, unilateral), Mental Health Act status, consent status, baseline and exit MADRS scores, baseline and exit CGI-S scores, and reported side effects.

A range of specific side effects were recorded including anesthetic complications, cardiovascular complications, cerebrovascular complications, headache, manic switch, muscle aches, nausea, and prolonged seizure. Cognitive side effects including confusion and self-reported memory problems were also recorded. A side effect was recorded as being present if the side effect was reported at any point during the episode that was being treated. For example, headache that occurred once after the second treatment of 10 was recorded as occurring during that course of treatment. Side effects were not mutually exclusive, and multiple side effects could be recorded.

Binary variables were used to indicate weekly (coded 0/1) or twice-weekly (coded 0/1) treatment; completion of an episode of ECT (coded 0/1); use of propofol, thiopentone, etomidate, suxamethonium, or other anesthetic agents (each medication coded as 0/1); and bilateral (0/1), bifrontal (0/1), and unilateral (0/1). For statistical analyses, a patient was considered capable of consent (coded 1) if T2 or informal was recorded (with T3A Part A, T3A Part B, S48, and urgent being considered incapable, coded as 0). Zero was considered as the reference category throughout. A T2 is completed when an individual is detained under the Mental Health (Care and Treatment) (Scotland) Act 2003 but is capable of providing informed consent for ECT treatment. A T3A Part A is completed when an individual is detained under the Mental Health (Care and Treatment) (Scotland) Act 2003 and is incapable of providing informed consent for treatment but is not resisting treatment. A T3A Part B is completed when an individual is detained under the Mental Health (Care and Treatment) (Scotland) Act 2003 and is incapable of providing informed consent to treatment but is resisting treatment.

Diagnoses were recorded using ICD-10 codes (18). The following codes were used to define indication for ECT: depressive disorder (F20.4, F32.X, F33.X, or F41.2); bipolar depression (F31.3, F31.4, or F31.5), mania (F30, F30.2, F31.1, or F31.2), schizophrenia (F20, F20.2, F20.9, or F23.1), schizoaffective disorder (F25, F25.0, F25.1, F25.2, or F25.9), mixed affective state (F31.6), personality disorder (F60, F60.3, and F60.9), postpartum disorders (F53, F53.0, F53.1, or F53.9), and other (F00, F06, F06.1, F06.3, F06.9, F22.0, F23, F29, F29.X, F31, F31.7, F31.8, F31.9, F34.0, F34.1, F34.8, F34.9, F38.0, F38.1, F38.8, F39, F40, F41, F41.1, F42, F43, F44, F44.9, F45. F45.2, F45.3, F50, F50.9, and Z.004). Each diagnosis was recorded as not present/present (coded 0/1), and diagnoses were mutually exclusive. Depressive disorder was considered as the reference category for all analyses.

### Statistical Analyses

#### Change in Symptom Severity

To explore change in symptom severity, entry and exit CGI-S scores were first compared using paired *t* tests, both in the total sample and by diagnosis. For depression and bipolar depression, entry and exit MADRS scores were also compared using paired *t* tests. Rates of side effects of ECT were compared across different diagnostic groups.

Subsequently, change in CGI-S scores (ΔCGI-S) were computed (Δ = entry score − exit score, so that a positive score reflects improvement, and a negative score reflects worsening of symptoms). ECT response was defined as ΔCGI-S ≥ 2. When data were missing for CGI-S at entry or exit, the individual was excluded from the analyses. Change in MADRS scores was computed as percentage (%) change. ECT response was defined as ΔMADRS ≥ 50% (19). Spearman’s rank correlation coefficients were calculated between CGI-S and MADRS scores at entry and exit, and between the change scores for each measure.

#### Which Factors Influenced ECT Response?

Logistic regression was used to explore whether individual or treatment variables influenced ECT response (ΔCGI-S ≥ 2). A minimally adjusted model was explored first, which included treatment episode completion (defined by the treating clinician), diagnosis (with depression as the reference), age at episode start, sex, CGI-S score at entry, and capability for consent. A fully adjusted model, including the minimal model plus episode year (2009 as reference); total treatment dose; maximum stimulations per treatment; use of propofol, thiopentone, etomidate, suxamethonium, or other muscle relaxant; and treatment schedule (weekly, twice weekly) was also explored. Analyses were restricted to participants who had up to 12 treatments per episode because this is the standard protocol (20).

Several sensitivity analyses were conducted. Firstly, for diagnoses of depression or bipolar depression, MADRS scores were considered, with ECT response defined as ΔMADRS ≥ 50% and the MADRS score at entry replacing the CGI-S score at entry as a covariate. Secondly, analyses also explored the impact of the number of treatments per episode. Twelve treatments is the standard regime (20); however, 63.3% of the episodes had ≤10 treatments, 83.2% had ≤12 treatments, and 96.1% had ≤18 treatments. Therefore, analyses considered ≤10 treatments and ≤18 treatments. Episodes with >18 treatments were likely to be maintenance therapy, so they were excluded from analyses.

#### Which Factors Influenced ECT Side Effects?

To explore whether individual or treatment variables influenced ECT side effects, logistic regression was used. As above, a minimally adjusted model was explored first, including treatment episode completion (defined by the treating clinician), diagnosis (with depression as the reference), age at episode start, sex, CGI-S score at entry, and capability for consent. A fully adjusted model that included the minimal model plus episode year (2009 as reference); total treatment dose; maximum stimulations per treatment; use of propofol, thiopentone, etomidate, suxamethonium, or other muscle relaxant; and treatment schedule (weekly, twice weekly) was also explored. Analyses were restricted to participants who had up to 12 treatments per episode.

All analyses were conducted using STATA 18.0 (21). False discovery rate (FDR) correction was used to account for multiple testing (22). A *p*_FDR_ < .05 was considered significant for these exploratory analyses.

## Results

### SEAN Data

A total of 4826 ECT episodes were recorded between 2009 and 2019, most of which were in females (68.4%, *n* = 3301 episodes) (Table S1). The average age at treatment onset was 58.5 years (SD 16.0 years). There was no significant difference in age between males and females (females = 58.7 years vs. males = 58.2 years, *p* = .20). The mean number of treatments per episode was 9.59 (95% CI, 9.32–9.85). There was no significant difference by sex in the mean number of treatments received per episode (females = 9.42, males = 9.93, *p* = .96). The mean treatment dose delivered was 277.75 mC (95% CI, 272.88–282.63 mC). Most treatments were completed as planned (68%, *n* = 3301).

Nearly all episodes (96%, *n* = 4633) had an ICD-10 diagnosis recorded. The most common diagnosis was depression (77.2%), followed by bipolar depression (10.9%). Other indications included mania (1.8%), schizophrenia (4.4%), schizoaffective disorder (2.1%), mixed affective disorder (0.8%), postpartum disorder (0.6%), and other (2.2%), which is a highly heterogeneous group. The sex distribution of the participants included in this study is presented in Table 1.

**Table 1:** Use of Electroconvulsive Therapy by ICD-10 Diagnosis

	Number of Episodes, *n* (%)	Women, *n* (%)	Men, *n* (%)	Age, Mean [IQR]	Treatments, Mean (SD)	Dose, mC, Mean [IQR]	Weekly, *n* (%)	Twice Weekly, *n* (%)	Treatment Completed, *n* (%)	Informal Status, *n* (%)
All	4633 (100%)	3172 (68.5%)	1461 (31.5%)	58.6 [48.0–71.0]	9.6 (9.4)	3239 [1194–3825]	90 (1.9%)	3034 (62.8%)	3198 (69.0%)	2957 (63.8%)
Depression	3577 (77.2%)	2454 (77.4%)	1123 (76.9%)	60.4 [49.0–73.0]	9.6 (9.8)	3213 [1175–3750]	62 (1.7%)	2276 (63.6%)	2500 (69.9%)	2229 (62.3%)
Bipolar Depression	506 (10.9%)	366 (11.5%)	140 (9.6%)	55.7 [46.0–67.0]	9.6 (9.5)	3564 [1323–4339]	15 (3.0%)	301 (59.5%)	326 (64.4%)	303 (59.9%)
Mania	83 (1.8%)	62 (2.0%)	21 (1.4%)	55.7 [48.0–65.0]	9.6 (6.7)	2565 [1000–2872]	1 (1.2%)	60 (72.3%)	57 (68.7%)	83 (100%)
Schizophrenia	204 (4.4%)	93 (2.9%)	111 (7.6%)	45.2 [35.0–56.0]	8.9 (4.3)	2838 [1217–3723]	2 (1.0%)	115 (56.4%)	115 (56.4%)	79 (38.7%)
Schizoaffective Disorder	97 (2.1%)	59 (1.9%)	38 (2.6%)	54.5 [45.0–66.5]	9.0 (5.8)	3166 [1230–3775]	2 (2.1%)	68 (70.1%)	76 (78.4%)	97 (100%)
Mixed Affective Disorder	38 (0.8%)	30 (1.0%)	8 (0.6%)	58.1 [15.3]	9.6 (12.3)	4771 [1000–4625]	3 (7.9%)	21 (55.3%)	26 (68.4%)	38 (100%)
Postpartum Disorders	27 (0.6%)	27 (1.0%)	0 (0%)	29.9 [6.8]	9.4 (3.2)	2913 [1100–4175]	0 (0%)	21 (77.8%)	19 (70.4%)	27 (100%)
Other	101 (2.2%)	81 (2.6%)	20 (1.4%)	51.3 [18.5]	9.1 (6.2)	3483 [1400–3987]	1 (1.0%)	81 (80.2%)	79 (78.2%)	101 (100%)

For the Women and Men columns, percentages are calculated based on total number of episodes for that sex. Percentages in all other columns are calculated based on total number of episodes for that diagnosis.

Overall, most recipients (62.4%, *n* = 2892) who received ECT were classified as informal, i.e., not detained under the Mental Health (Care and Treatment) (Scotland) Act (Table 1). However, rates of informal treatment varied by diagnosis, being lowest for schizophrenia (17.7%, *n* = 36) followed by mania (22.9%, *n* = 19).

### Change in Symptom Severity

A total of 2920 episodes of treatment had both CGI-S entry and exit information available (Table 2). At entry, recipients were markedly ill based on the CGI-S scores (5.0; 95% CI, 5.0–5.1). Overall, there was improvement in symptoms over time, with recipients being borderline ill at exit (CGI-S, 2.1; 95% CI, 2.0–2.1), which was statistically significant for all diagnoses. Recipients with a schizophrenia diagnosis had the highest entry CGI-S score (5.45; 95% CI, 5.21–5.60), followed by the heterogeneous other diagnoses group (5.05; 95% CI, 4.68–5.41). The lowest exit CGI-S scores were seen in recipients with a mixed affective state (1.72; 95% CI, 0.99–2.47) followed by recipients with schizoaffective disorder (2.01; 95% CI, 1.76–2.42).

**Table 2:** CGI-S and MADRS Scores at Entry and Exit by Diagnosis

Diagnosis	*n*	CGI-S			MADRS		
		Entry, Mean (SD)	Exit, Mean (SD)	*p*_FDR_	Entry, Mean (SD)	Exit, Mean (SD)	*p*_FDR_
All	2837	5.03 (1.13)	2.07 (1.13)	<.0001^a^	–	–	–
Depression + Bipolar Depression	2619	–	–	–	37.13 (10.80)	13.39 (11.63)	<.0001^a^
Depression	2249	5.00 (1.10)	2.06 (1.13)	<.0001^a^	37.17 (10.83)	13.46 (11.72)	<.0001^a^
Bipolar Depression	302	5.02 (1.08)	2.02 (1.08)	<.0001^a^	36.79 (10.56)	12.91 (10.98)	<.0001^a^
Mania	42	5.40 (1.08)	2.26 (0.99)	<.0001^a^	–	–	–
Schizophrenia	110	5.45 (1.28)	2.19 (1.12)	<.0001^a^	–	–	–
Schizoaffective Disorder	68	4.94 (1.54)	2.01 (1.36)	<.0001^a^	–	–	–
Mixed Affective Disorder	15	4.67 (1.68)	1.73 (1.33)	<.0001^a^	–	–	–
Postpartum Disorders	8	5.38 (0.92)	2.13 (0.99)	<.0001^a^	–	–	–
Other	43	5.05 (1.19)	2.30 (1.30)	<.0001^a^	–	–	–

CGI-S: 1 = normal, not it ill at all, 2 = borderline mentally ill, 3 = mildly ill, 4 = moderately ill, 5 = markedly ill, 6 = severely ill, and 7 = extremely ill. MADRS, ≤10 = no depression (or remission), 10−30 = depression, and >30 = severe depression.

CGI-S, Clinical Global Impression-Severity; MADRS, Montgomery–Åsberg Depression Rating Scale; *p*_FDR_, false discovery rate–corrected *p*.

a Statistically significant.

In the depression and bipolar depression group, 2619 episodes of treatment had both MADRS entry and exit information available (Table 2). For recipients with depression or bipolar depression, the mean MADRS score at entry was within the depressed range (37.18; 95% CI, 36.73–37.61 or 36.79; 95% CI, 35.60–37.98, respectively). At exit, mean MADRS scores, although still above the no depression/remission threshold (≤10), were significantly lower (13.46; 95% CI, 12.98–13.94 and 12.91; 95% CI, 11.68–14.15, respectively).

**Figure 1:**
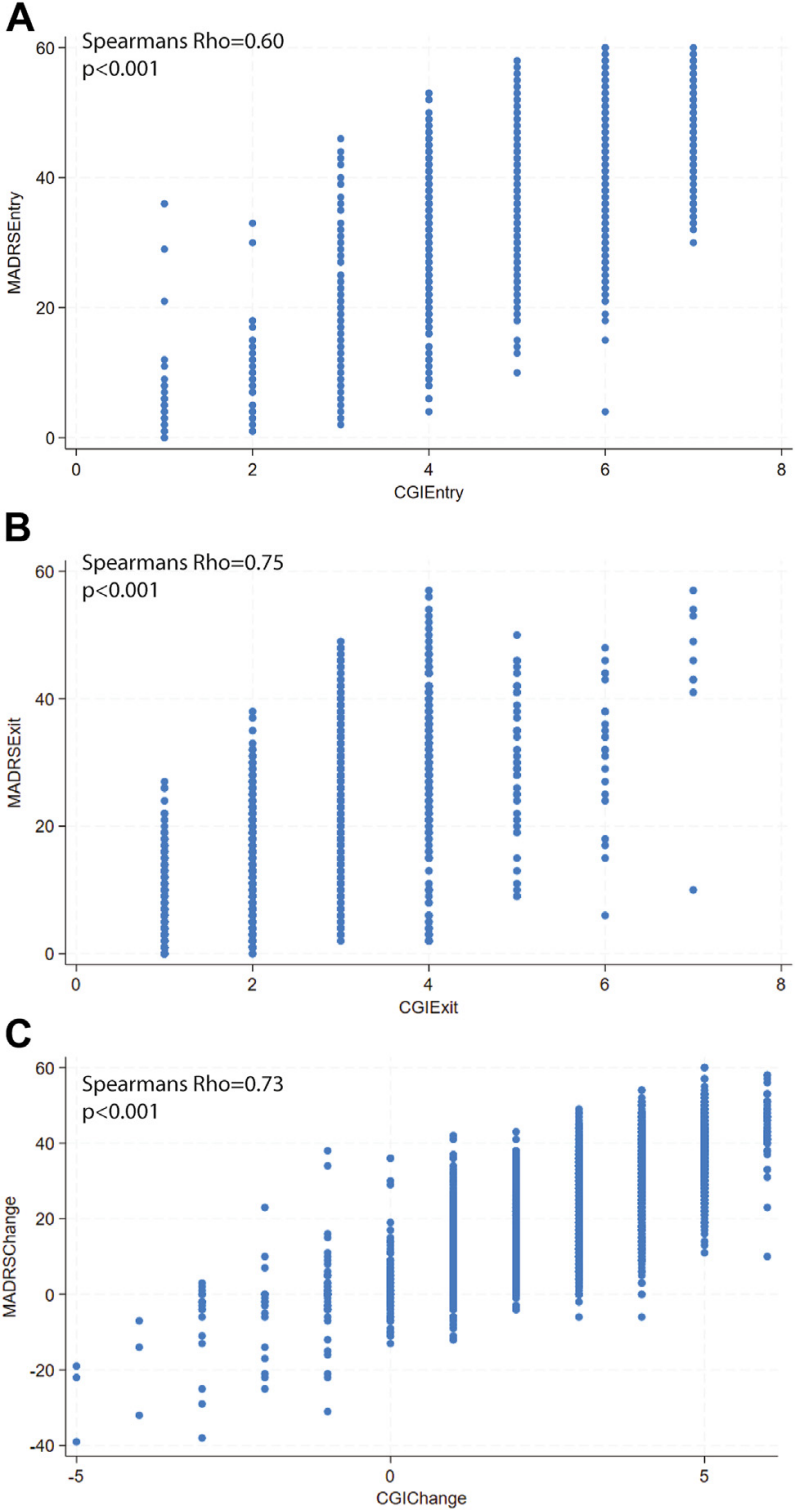
Spearman’s rank correlations between Clinical Global Impression (CGI)-Severity scale and Montgomery–Åsberg Depression Rating Scale (MADRS) scores **(A)** at entry, **(B)** at exit, and **(C)** change in scores.

**Figure 2:**
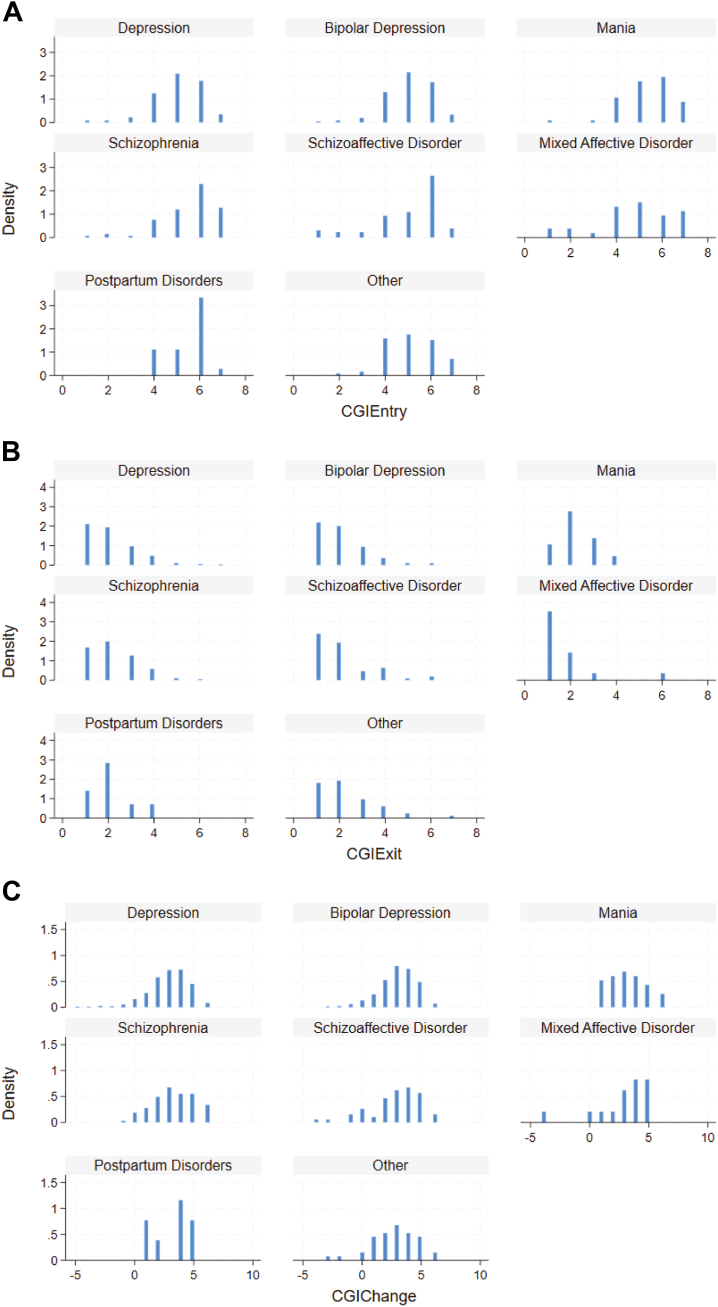
Distribution of Clinical Global Impression (CGI)-Severity scale scores by diagnoses at **(A)** entry, **(B)** exit, and **(C)** change. Individuals with up to 12 treatments per episode were included. For CGI-Severity change, positive values indicate improvement, and negative values indicate worsening.

**Figure 3:**
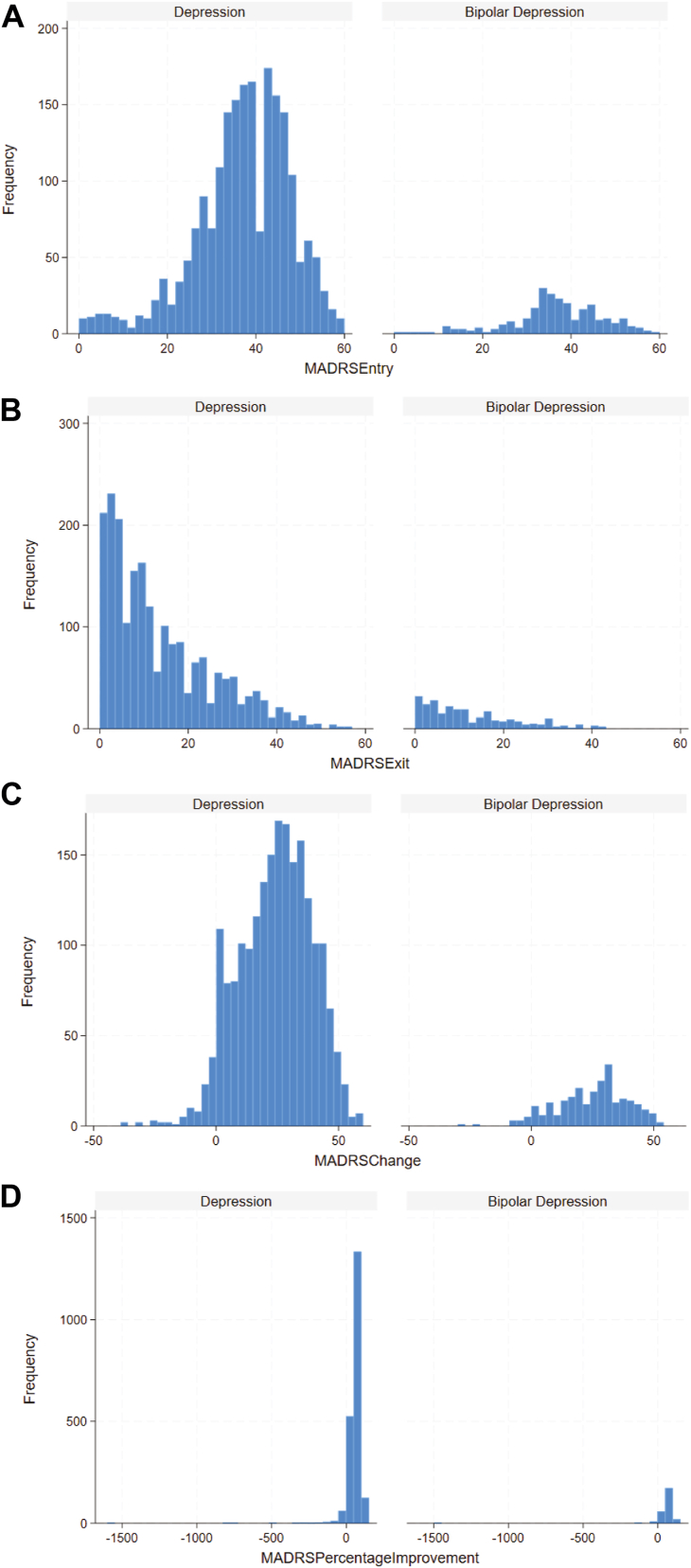
Distribution of Montgomery–Åsberg Depression Rating Scale (MADRS) scores by diagnoses at **(A)** entry, **(B)** exit, **(C)** change in scores, and **(D)** percentage change in scores. Individuals with up to 12 treatments per episode were included.

Strong correlations were observed between ΔCGI-S and CGI-S scores at entry (ρ = 0.75, *p* < .001) and exit (ρ = −0.74, *p* < .001). Strong correlations were also observed between ΔMADRS and MADRS scores at entry (ρ = 0.60, *p* < .001) and exit (ρ = −0.67, *p* < .001) for recipients with depression or bipolar depression. Among recipients with depression or bipolar depression who had both CGI-S and MADRS scores (*n* = 2495), CGI scores demonstrated strong positive correlations (0.60 for entry, 0.75 for exit, and 0.73 for change; all *p*s < .001) (Figure 1). The distributions at entry, exit, and change in CGI-S scores are presented in Figure 2. Similarly, the distributions for entry, exit, and percentage change in MADRS scores for recipients with depression or bipolar depression are presented in Figure 3.

### Variables That Influenced ECT Response (ΔCGI-S ≥ 2 or ΔMADRS ≥ 50%)

We observed 79% to 83% of treatment episodes with ECT response based on CGI-S scores and 81% to 83% with ECT response based on MADRS scores (Table 3).

**Table 3:** Electroconvulsive Therapy Response by Diagnosis

Diagnosis	*n*	ΔCGI		CGI-S at Exit = 1	CGI-S at Exit = 2	*n*	ΔMADRS		MADRS at Exit ≤ 10
		≥2	<2				≥50%	<50%	
All Diagnoses	2837	2341 (82.5%)	496 (17.5%)	1091 (38.5%)	1007 (35.5%)	4083	3306 (81.0%)	777 (19.0%)	1523 (37.3%)
Depression	2249	1860 (82.7%)	389 (17.3%)	851 (37.8%)	768 (34.2%)	3577	2887 (80.7%)	690 (19.3%)	1206 (33.7%)
Bipolar Depression	302	252 (83.4%)	50 (16.6%)	115 (38.1%)	109 (36.1%)	506	419 (82.8%)	87 (17.2%)	159 (31.4%)
Mania	42	34 (81.0%)	8 (19.1%)	9 (21.4%)	19 (45.2%)		–	–	–
Schizophrenia	110	90 (81.8%)	20 (18.2%)	35 (31.8%)	38 (34.6%)		–	–	–
Schizoaffective Disorder	68	54 (79.4%)	14 (20.6%)	29 (42.7%)	23 (33.8%)		–	–	–
Mixed Affective Disorder	15	12 (80.0%)	3 (20.0%)	9 (60.0%)	4 (26.7%)		–	–	–
Postpartum Disorders	8	6 (75.0%)	2 (25.0%)	2 (25.0%)	4 (50.0%)		–	–	–
Other	43	33 (76.7%)	10 (23.3%)	13 (30.2%)	15 (34.9%)		–	–	–

Values are presented as *n* (%). ΔCGI > 0 was considered an improvement, ΔCGI = 0 was considered neutral, and ΔCGI < 0 was considered worsened. ΔMADRS > 50% was considered an improvement, 0% to 49.9% was considered neutral, and <50% was considered worsened.

CGI-S, Clinical Global Impression-Severity; MADRS, Montgomery–Åsberg Depression Rating Scale.

When investigating variables that influence ECT response as measured by change in CGI-S scores (ΔCGI-S ≥ 2) (Table 4), the minimal model included 2528 individuals and highlighted that increased age (odds ratio 1.01; 95% CI, 1.01–2.03; *p*_FDR_ = .006) and increased CGI-S score at entry (4.62; 95% CI, 3.95–5.40; *p*_FDR_ = .001) were positively associated. The results were not changed in the fully adjusted model or any further significant variables identified. Similar results were identified when MADRS scores were used (Table 4); however, female sex was also positively associated with ECT response (odds ratio 1.26; 95% CI, 1.03–1.53; *p*_FDR_ = .042) in the minimally adjusted model but did not survive multiple testing correction in the fully adjusted model.

**Table 4:** Variables That Influenced Electroconvulsive Therapy Effectiveness

	Effective Based on ΔCGI ≥ 2, *n* = 2528				Effective Based on ΔMADRS ≥50%, *n* = 2332			
	Minimally Adjusted		Fully Adjusted		Minimally Adjusted		Fully Adjusted	
	OR (95% CI)	*p*_FDR_	OR (95% CI)	*p*_FDR_	OR (95% CI)	*p*_FDR_	OR (95% CI)	*p*_FDR_
Episode Complete	0.79 (0.60–1.04)	.288	0.85 (0.52–1.41)	.854	0.84 (0.68–1.03)	.132	0.82 (0.57–1.19)	.564
Bipolar Depression	1.23 (0.81–1.89)	.545	1.27 (0.82–1.97)	.840	1.22 (0.90–1.64)	.239	1.30 (0.95–1.78)	.485
Mania	0.69 (0.24–1.93)	.570	0.68 (0.24–1.94)	.841	–	–	–	–
Schizophrenia	0.80 (0.40–1.60)	.579	0.86 (0.42–1.74)	.854	–	–	–	–
Schizoaffective Disorder	1.63 (0.62–4.30)	.545	1.72 (0.64–4.62)	.840	–	–	–	–
Mixed Affective Disorder	2.06 (0.34–12.54)	.570	2.12 (0.36–12.48)	.840	–	–	–	–
Postpartum Disorders	0.40 (0.06–2.84)	.545	0.34 (0.05–2.40)	.840	–	–	–	–
Other Diagnosis	0.61 (0.26–1.46)	.545	0.62 (0.25–1.52)	.840	–	–	–	–
Age	1.01 (1.01–1.02)	.006^a^	1.01 (1.01–1.02)	.017^a^	1.02 (1.01–1.03)	<.0001^a^	1.01 (1.01–1.02)	<.0001^a^
Sex, Female	1.31 (1.01–1.70)	.184	1.33 (1.03–1.73)	.419	1.26 (1.03–1.53)	.042^a^	1.21 (0.99–1.49)	.485
CGI/MADRS at Entry	4.62 (3.95–5.40)	.001^a^	4.75 (4.05–5.56)	<.0001^a^	1.05 (1.04–1.06)	<.0001^a^	1.50 (1.37– 1.64)	<.0001^a^
Consent Status	0.98 (0.74–1.29)	.881	1.04 (0.79–1.38)	.854	0.89 (0.73–1.08)	.239	0.88 (0.72–1.08)	.517
2010	–	–	1.08 (0.62–1.86)	.854	–	–	0.94 (0.62–1.44)	.928
2011	–	–	1.05 (0.59–1.88)	.885	–	–	0.82 (0.52–1.27)	.621
2012	–	–	0.60 (0.35–1.04)	.587	–	–	0.75 (0.49–1.16)	.509
2013	–	–	1.35 (0.77–2.36)	.840	–	–	0.74 (0.48–1.14)	.509
2014	–	–	0.77 (0.44–1.33)	.840	–	–	0.86 (0.56–1.34)	.803
2015	–	–	1.25 (0.69–2.26)	.841	–	–	0.96 (0.61–1.51)	.928
2016	–	–	1.35 (0.75–2.45)	.840	–	–	1.29 (0.80–2.06)	.564
2017	–	–	1.16 (0.62–2.16)	.854	–	–	0.94 (0.59–1.51)	.928
2018	–	–	1.35 (0.74–2.47)	.840	–	–	1.04 (0.66–1.64)	.928
2019	–	–	1.21 (0.65–2.25)	.854	–	–	0.73 (0.46–1.15)	.509
Electrode Placement: Bilateral	–	–	1.13 (0.48–2.66)	.854	–	–	1.08 (0.54–2.19)	.928
Electrode Placement: Bifrontal	–	–	0.63 (0.28–1.42)	.840	–	–	1.13 (0.57–2.26)	.928
Electrode Placement: Unilateral	–	–	0.64 (0.35–1.17)	.840	–	–	0.88 (0.54–1.44)	.906
Propofol^b^	–	–	0.94 (0.58–1.53)	.854	–	–	0.75 (0.51–1.09)	.509
Thiopentone^b^	–	–	0.87 (0.54–1.38)	.854	–	–	1.38 (0.94–2.02)	.485
Etomidate^b^	–	–	0.81 (0.51–1.31)	.840	–	–	0.79 (0.55–1.13)	.509
Suxamethonium^b^	–	–	1.40 (0.13–15.09)	.854	–	–	0.86 (0.08–9.78)	.937
Other Muscle Relaxant^b^	–	–	1.50 (0.75–3.00)	.840	–	–	1.38 (0.81–2.35)	.517
Treatment Dose Total	–	–	1.00 (1.00 = 1.00)	.854	–	–	1.00 (1.00–1.00)	.621
Maximum Stimulations per Treatment	–	–	0.87 (0.75–1.01)	.854	–	–	0.91 (0.82–1.02)	.485
Weekly Treatment	–	–	0.99 (0.37–2.67)	.982	–	–	0.89 (0.40–1.95)	.928
Twice-Weekly Treatment	–	–	0.88 (0.55–1.41)	.854	–	–	1.01 (0.71–1.43)	.973

Reference episode year = 2009; reference diagnosis = depression. Treatment episodes up to 12 included. Notably, electrode placements are not mutually exclusive because treatment might have started with one placement before being switched to a different placement.

CGI, Clinical Global Impressions; MADRS, Montgomery–Åsberg Depression Rating Scale; OR, odds ratio; *p*_FDR_, false discovery rate–corrected *p*.

a Statistically significant.

b Presence of (binary).

Sensitivity analyses demonstrated similar results when ECT response was defined as ΔCGI-S ≥ 4 (Table S2) and when the number of treatments per episode was ≤10 or ≤18 (Table S3).

It is worth noting that recipients who did not attend the final scheduled treatment did not complete the CGI-S or MADRS at exit and therefore were excluded from these analyses. Individuals without a completed CGI-S at exit had higher CGI-S scores at entry than those who completed treatment (mean [SD] 5.15 [1.18] vs. 5.03 [1.13], *t* test *p* = .0011); however, there was no significant difference in MADRS scores at entry in recipients with and without a completed MADRS at exit (37.06 [11.28] vs. 37.13 [10.80], respectively, *t* test *p* = .862).

### Variables That Influenced Side Effects

Anesthetic complications and prolonged seizures were rare, occurring in <1% of treatment episodes (Table S4). A reported manic switch was also relatively rare, occurring in just over 1% of treatment episodes (Table S4). Cardiovascular complications were reported in 2.2% of treatment episodes. Nausea was more common (7.4%), as were muscle aches (12%). Confusion and acute confusion were reported in 15% and 6% of treatment episodes, respectively, and cognitive side effects or memory problems were reported in 13% and 18%, respectively.

The impact of individual and treatment variables on side effects is summarized in Table S5. A diagnosis of schizophrenia was negatively associated with self-reported headaches (0.34; 95% CI, 0.22–0.55; *p*_FDR_ < .0001) and memory problems (0.43; 95% CI, 0.25–0.75; *p*_FDR_ = .026). A diagnosis of schizoaffective disorder was negatively associated with self-reported memory problems (0.25; 95% CI, 0.14–0.91; *p*_FDR_ = .027). Increased age was associated with reduced risk of self-reported headaches, memory problems, muscle aches, and nausea (Table S5). Female sex was associated with increased risk of self-reported memory and cognitive problems (Table S5). Increased CGI-S score at entry (i.e., more severely ill) was associated with reduced risk of memory and cognitive problems and muscle aches (Table S5). Being capable of consenting was associated with increased risk of cognitive problems and muscle aches. The anesthesia used was associated with an increased risk of side effects including cognitive problems and confusion (propofol) as well as headaches (thiopentone) (Table S5). The maximum number of stimulations was negatively associated with risk of cognitive problems, and twice-weekly treatment was associated with reduced risk of cognitive problems (Table S5).

## Discussion

In this study, we report on response to and side effects of ECT using the SEAN dataset, which included 4826 ECT episodes. Our findings provide evidence that a response to ECT is reported in a range of diagnoses, that cognitive problems and confusion are the most common side effects, and that other side effects are less common (<12%). Furthermore, age and illness severity appear to be associated with both improved response to ECT and less side effects.

Previous studies have reported the effectiveness of ECT for schizophrenia (4), mania (23), postpartum psychosis (24), and major depression (25). Our results in the Scottish population are consistent with these reports and further demonstrate that ECT is beneficial for a wide range of diagnoses including bipolar disorder and affective disorders. For nonemergency cases, ECT is generally considered after there has been a limited response to multiple other therapies; therefore, this patient group can be considered treatment resistant. In this context, a response rate of >82% is particularly striking and highlights the essential role of ECT in clinical practice. It is also worth noting that increased symptom severity (either CGI-S or MADRS scores) was positively associated with response and negatively associated with side effects, suggesting that the most severely ill benefited the most. The association of increased age (for depression and bipolar depression) with response to ECT observed here is consistent with previous reports (25). However, we cannot exclude the possibility that this finding is driven by the demographics of the individuals included in the study. ECT is typically only used after multiple medications have failed to achieve the desired response (26). Consequently, the recipients included in this study were on the older end of the spectrum of age of people with these psychiatric conditions.

In our study, there appeared to be no difference in response to treatment whether ECT was given once weekly or twice weekly. This is somewhat surprising because twice-weekly ECT is usually recommended in acute courses (27). Notably, the number of overall treatment episodes that occurred weekly was low (1.8%). Therefore, we cannot exclude the possibility that the treatment schedule includes consideration of tolerance and preexisting frailty or cognitive problems. Low statistical power could also explain the null effect. Regarding the impact of the treatment schedule on cognition, it has previously been shown that twice-weekly treatment was associated with lesser short-term cognitive impairment than three times weekly (28). Here, we included weekly and twice-weekly treatment, with other schedule as the reference category. Therefore, our findings are consistent with previous research. That said, we cannot exclude the possibility that the treatment schedule includes consideration of tolerance and preexisting frailty or cognitive problems.

In terms of the associations of diagnoses with side effects, the small sample size for most groups limits statistical power, and therefore these findings may not be reproducible. In terms of cardiovascular side effects, as mentioned above, ECT is generally considered after there has been a limited response to various other treatments (26), and it is likely that recipients undergoing ECT have previously been prescribed a number of antidepressant medications. Therefore, the increased age of these recipients (compared with the average age of individuals with these diagnoses) is associated with increased risk of cardiovascular disease. In addition, many antidepressants have adverse cardiometabolic effects; therefore, the treatments previously attempted in these recipients are also associated with increased risk of cardiovascular side effects (although this association did not survive multiple testing correction). We were unable to directly assess this hypothesis with these data; however, this would be of interest to explore in the future. The impact of treatment variables such as the anesthetic used (a clinical decision based on patient characteristics during ECT) and treatment dose and timing (weekly vs. twice weekly) on risk of self-reported confusion and cognitive problems suggest that this area warrants further investigation. The relative frequency of cognitive problems and confusion reported is important to consider in the context of the psychiatric disorders being treated. Cognitive problems are often reported together with psychiatric disorders, particularly depression, and may persist during periods of illness remission (29,30). It has previously been shown that increased age was associated with a lesser impact of ECT on cognition (7) in individuals with an average age of 45.7 (16.9) years, which is consistent with the lack of impact in our older population (58.5 [16] years). Previous studies have reported the impact of the tool used to assess cognitive impact (31).

SEAN data collection continues, with more comprehensive data on cognition being collected now, so reporting of more detailed information on cognition should be possible in the future. It may also be possible to conduct studies with linkage to other national electronic records, which would allow investigation into the impact of additional clinical factors (such as previous and current treatment modalities) on ECT response and side effects (including longer-term physical comorbidities).

Although there is a robust correlation between CGI-S and MADRS scores when measuring antidepressant response (32), there has been some uncertainty regarding the utility of the CGI-S for measuring response to ECT. A study by Chen *et al.* (33) of over 1100 patients explored response to ECT using a range of clinical outcome measures including the MADRS, Brief Psychiatric Rating Scale, and CGI-S. They found no significant difference in average total Brief Psychiatric Rating Scale or CGI-S scores. Overall, the consistency in findings between (change in) MADRS and CGI-S scores gives us confidence that CGI-S is an appropriate tool for assessing response to ECT. CGI is recognized as a valid clinical outcome measure for use in psychiatric research (34).

Despite this being one of the largest studies of ECT reported to date, there are several important limitations. First, while the data cover 4826 treatment episodes, only 2484 (51%) had complete entry and exit CGI-S scores. Second, we were unable to differentiate between individuals undergoing single treatment episodes, repeated treatment episodes, or maintenance treatment, and we do not know how many individuals are represented in this dataset. Information on titration of dose was also missing. A recent study highlighted the value of maintenance ECT (35); however, SEAN data did not include sufficient information on treatment timings to be able to conduct comparable analyses. Another recent study highlighted the impact of seizure duration and anticonvulsant medication on ECT effectiveness (36); however, these data were not available, so we were unable to attempt to validate these findings using the SEAN data. Prescribing information was unavailable, so while we believe that this population is treatment resistant, we are unable verify this. Similarly, we lack data on diagnostic comorbidity. However, by including individuals with ≤12 treatment episodes (main analysis, current National Institute for Health and Care Excellence guidelines), which represented 89% of the dataset, it is likely that we included acute courses of ECT only. We acknowledge that the “other” diagnostic group is highly heterogeneous and therefore not as informative as the results from well-defined diagnoses. That said, our results demonstrate that this heterogeneous group did benefit from ECT and therefore could be of relevance when considering treatment of patients with complex, unclear, and/or multiple diagnoses. Side effects were also recorded by the clinical team, and therefore there may be heterogeneity in reporting. Finally, the numbers of individuals in diagnostic groups other than depression, bipolar depression, and schizophrenia were small (*n* < 70), thereby limiting statistical power and robustness of findings. It should be further stressed that the results reported here are associations and therefore cannot be interpreted as causal effects.

### Conclusions

In a large study of ECT, we have demonstrated that response to ECT (measured by reduction in CGI severity or MADRS scores) occurs across a range of diagnoses. We also demonstrated that increased age and CGI severity or MADRS scores at entry were the most significant variables associated with treatment response. Overall, these results provide evidence of the efficacy and relative safety of ECT for a range of psychiatric diagnoses.

## Supplementary Material

Table S5

Key Resources Table
