# Supplementary material for: Electroconvulsive Therapy: A Scotland-Wide Naturalistic Study of 4826 Treatment Episodes
Source: Biol Psychiatry Glob Open Sci. 2024 Dec 16;5(2):100434. doi: 10.1016/j.bpsgos.2024.100434 (PMC11804586; doi:10.1016/j.bpsgos.2024.100434)
Supplement: Tables S1–S4 [file mmc1.pdf]

SUPPLEMENTARY INFORMATION

Electroconvulsive Therapy: A Scotland-Wide Naturalistic Study of 4,826 Treatment Episodes

Martin *et al.*

Supplementary Tables

Supplementary Table 1: Summary table of use of ECT

Supplementary Table 2: Sensitivity testing CGI threshold for response

Supplementary Table 3: Sensitivity testing number of treatments.

Supplementary Table 4: Side effects by diagnosis, in patients with ≤12 treatments.

Supplementary Table 5: Variables influencing side effects (See separate Excel file)

Supplementary Table 1: Summary table of use of ECT

		All	Men (n=1523)	Women (n=3298)	FDR P
Age at treatment onset	mean (sd)	58.5 (16.0)	58.2 (15.0)	58.7 (16.5)	0.606
	IQR	48-71	49-69	47-72	
SIMD	n	3325	1084	2241	
1-Most deprived	n (%)	709 (21.3)	238 (22.0)	471 (21.0)	
2	n (%)	729 (21.9)	208 (19.2)	521 (23.3)	
3	n (%)	663 (19.9)	238 (22.0)	425 (19.0)	
4	n (%)	688 (20.7)	219 (20.2)	469 (20.9)	
5- least deprived	n (%)	536 (12.1)	181 (16.7)	355 (15.8)	0.956
Number of treatments	mean (sd)	9.6 (9.4)	9.9 (9.7)	9.4 (9.2)	0.956
	IQR	6-12	6-12	6-12	
Mean Dose	mean (sd)	278 (173)	281 (176)	276 (171)	0.956
	IQR	158-350	161-351	156-350	
Electrode placement: bifrontal	n (%)	94 (2.0)	24 (1.6)	70 (2.1)	0.121
Electrode placement: bilateral	n (%)	4688 (97.1)	1483 (97.3)	3203 (97.0)	0.378
Electrode placement: unilateral	n (%)	356 (7.4)	101 (6.6)	255 (7.7)	0.096
Weekly treatment	n (%)	90 (1.9)	26 (1.7)	64 (1.9)	
Twice weekly treatment	n (%)	3034 (63.8)	931 (61.1)	2102 (63.7)	
Other schedule	n (%)	1707 (34.3)	568 (37.2)	1135 (34.4)	
Completed as planned	n (%)	3301 (68.4)	1020 (66.9)	2280 (69.1)	0.606
Informal treatment	n (%)	3028 (62.7)	945 (62.0)	2083 (63.1)	0.896

Where: sd, standard deviation; IQR, interquartile range. Of note, electrode placements are not mutually exclusive, as treatment might have started with one placement before being switched to a different placement.

Supplementary Table 2: Sensitivity testing CGI threshold for response

	Effective based on deltaCGI≥4, 12 treatments (N=2528)			
	minally adjusted		fully adjusted	
	OR (95% CI)	FDR P	OR (95% CI)	FDR P
episode complete	0.95 (0.75-1.19)	0.694	1.16 (0.76-1.77)	0.798
BD	1.12 (0.79-1.60)	0.636	1.12 (0.78-1.60)	0.798
Mania	0.51 (0.20-1.32)	0.279	0.53 (0.20-1.40)	0.618
SCZ	0.53 (0.30-0.95)	0.096	0.51 (0.28-0.93)	0.230
SAD	1.07 (0.50-2.30)	0.856	1.10 (0.51-2.36)	0.818
Mixed Affective Disorder	6.04 (1.24-29.45)	0.096	5.37 (1.03-28.09)	0.261
Postpartum Disorders	3.19 (0.41-24.59)	0.399	3.23 (0.39-27.06)	0.632
Other	0.73 (0.28-1.88)	0.636	0.73 (0.28-1.93)	0.798
age	1.02 (1.01-1.03)	**0.001**	1.02 (1.01-1.03)	**<0.0001**
sex (female)	1.27 (1.01-1.60)	0.101	1.28 (1.01-1.62)	0.261
CGI/MADRS at entry	9.19 (7.72-10.94)	**0.001**	9.39 (7.87-11.20)	**<0.0001**
Consent status	0.81 (0.65-1.02)	0.152	0.80 (0.63-1.00)	0.267
2010			0.77 (0.47-1.24)	0.632
2011			1.14 (0.69-1.90)	0.798
2012			1.11 (0.68-190)	0.814
2013			0.75 (0.46-1.24)	0.632
2014			0.71 (0.43-1.17)	0.602
2015			0.92 (0.55-1.53)	0.818
2016			0.93 (0.56-1.55)	0.818
2017			0.94 (0.56-1.58)	0.818
2018			0.93 (0.56-1.55)	0.818
2019			0.86 (0.51-1.45)	0.798
Electrode placement: bilateral			0.78 (0.33-1.87)	0.798
Electrode placement: bifrontal			0.79 (0.35-1.78)	0.798
Electrode placement: unilateral			0.66 (0.37-1.18)	0.602
propofol*			0.59 (0.39-0.90)	0.159

thiopentone*	0.94 (0.61-1.45)	0.818
etomidate*	0.74 (0.49-1.12)	0.602
suxamethonium*	0.52 (0.08-3.18)	0.798
otherMR*	1.18 (0.64-2.18)	0.798
treatment dose total	1.00 (1.00-1.00)	0.798
max stimulations per treatment	1.06 (0.93-1.20)	0.798
weekly treatment	1.22 (0.53-3.09)	0.814
twice weekly treatment	0.79 (0.28-1.93)	0.632

Where: FDR P, false discovery rate-corrected P; Reference episode year =2009; Reference diagnosis= depression. *prescence of (binary). Treatment episodes up to 12 included. Of note, electrode placements are not mutually exclusive, as treatment might have started with one placement before being switched to a different placement.

Supplementary Table 3: Sensitivity testing number of treatments.

	Effective based on deltaCGI≥2 up to 10 treaments (N=1773)				Effective based on deltaCGI≥2 up to 18 treaments (N=2725)			
	minimally adjusted		fully adjusted		minimally adjusted		fully adjusted	
	OR (95% CI)	FDR P	OR (95% CI)	FDR P	OR (95% CI)	FDR P	OR (95% CI)	FDR P
episode complete	0.63 (0.44-0.88)	**0.032**	0.62 (0.32-1.18)	0.486	0.83 (0.64-1.08)	0.422	0.95 (0.59-1.53)	0.938
BD	1.33 (0.80-2.23)	0.399	1.45 (0.85-2.48)	0.547	1.18 (0.79-1.77)	0.460	1.18 (0.78-1.78)	0.701
Mania	0.78 (0.23-2.65)	0.694	0.66 (0.19-2.31)	0.840	0.53 (0.21-1.33)	0.422	0.56 (0.22-1.43)	0.701
SCZ	0.80 (0.35-1.86)	0.664	0.98 (0.41-2.33)	0.979	0.71 (0.37-1.37)	0.460	0.76 (0.39-1.47)	0.701
SAD	2.39 (0.70-8.13)	0.326	2.75 (0.78-9.69)	0.468	1.51 (0.60-3.77)	0.460	1.56 (0.61-3.95)	0.701
Mixed Affective Disorder	2.26 (0.36-14.29)	0.463	2.60 (0.43-15.56)	0.699	2.09 (0.35-12.54)	0.460	2.16 (0.37-12.61)	0.701
Postpartum Disorders	0.25 (0.02-2.61)	0.399	0.27 (0.02-2.98)	0.699	0.42 (0.06-2.91)	0.460	0.36 (0.05-2.47)	0.701
Other	0.59 (0.21-1.60)	0.399	0.64 (0.22-1.87)	0.824	0.63 (0.26-1.49)	0.460	0.64 (0.26-1.55)	0.701
age	1.02 (1.01-1.02)	**0.018**	1.01 (1.00-1.02)	0.068	1.01 (1.01-1.02)	**0.001**	1.01 (1.01-1.02)	**0.017**
sex (female)	1.31 (0.96-1.80)	0.279	1.34 (0.97-1.86)	0.398	1.25 (0.97-1.61)	0.312	1.27 (0.99-1.64)	0.544
CGI/MADRS at entry	4.88 (4.02-5.91)	**0.001**	5.14 (4.21-6.27)	**<0.0001**	4.51 (3.90-5.23)	**0.001**	4.63 (3.99-5.38)	**<0.0001**
Consent status	0.78 (0.55-1.09)	0.326	0.84 (0.44-3.39)	0.699	0.96 (0.74-1.25)	0.749	1.00 (0.76-1.31)	0.990
2010			1.21 (0.63-2.30)	0.872			1.08 (0.64-1.83)	0.938
2011			1.07 (0.54-2.11)	0.954			1.05 (0.60-1.85)	0.938
2012			0.51 (0.27-0.97)	0.272			0.61 (0.36-1.03)	0.544
2013			1.16 (0.60-2.22)	0.872			1.18 (0.69-2.03)	0.765
2014			0.84 (0.44-1.61)	0.872			0.79 (0.46-1.34)	0.701
2015			0.95 (0.47-1.94)	0.979			1.37 (0.77-2.44)	0.701
2016			1.60 (0.76-3.36)	0.604			1.20 (0.68-2.11)	0.765
2017			1.03 (0.49-2.18)	0.979			0.98 (0.55-1.76)	0.990
2018			2.02 (0.91-4.46)	0.398			1.37 (0.76-2.45)	0.701
2019			1.38 (0.62-3.07)	0.824			1.17 (0.65-2.11)	0.805
Electrode placement: bilateral			1.23 (0.44-3.39)	0.875			1.09 (0.48-2.49)	0.938
Electrode placement: bifrontal			0.71 (0.25-2.00)	0.840			0.53 (0.25-1.10)	0.612
Electrode placement: unilateral			0.57 (0.28-1.17)	0.468			0.65 (0.37-1.16)	0.701

propofol*	0.73 (0.40-1.34)	0.699	0.92 (0.59-1.46)	0.938
thiopentone*	0.51 (0.29-0.89)	0.193	0.86 (0.55-1.33)	0.762
etomidate*	0.91 (0.51-1.64)	0.896	0.79 (0.50-1.23)	0.701
suxamethonium*	1.79 (0.13-24.47)	0.872	2.34 (0.38-14.54)	0.701
other MR*	1.32 (0.58-3.00)	0.840	1.35 (0.71-2.56)	0.701
treatment dose total	1.00 (1.00-1.00)	0.872	1.00 (1.00-1.00)	0.938
max stimulations per treatment	0.82 (0.68-0.99)	0.272	0.90 (0.79-1.04)	0.701
weekly treatment	1.21 (0.37-3.89)	0.896	1.02 (0.40-2.57)	0.990
twice weekly treatment	1.01 (0.54-1.88)	0.979	0.83 (0.53-1.30)	0.701

Where: FDR P, false discovery rate-corrected P; Reference episode year =2009; Reference diagnosis= depression. *presence of (binary). Treatment episodes up to 12 included. Of note, electrode placements are not mutually exclusive, as treatment might have started with one placement before being switched to a different placement.

Supplementary Table 4: Side effects by Diagnosis, in patients with ≤12 treatments.

ICD 10 Diagnosis	Headaches n (%)	Memory n (%)	Confusion n (%)	Cognitive Side Effect n (%)	Muscle Aches n (%)	Nausea n (%)	Acute confusion n (%)	Cardio-vascular n (%)	Manic switch n (%)	Cerebro-vascular n (%)	Prolonged Seizure n (%)	Anaesthetic complications n (%)	Other n (%)
All (n=4633)	1197 (29.0)	732 (17.7)	634 (15.3)	546 (13.2)	503 (12.2)	305 (7.4)	241 (5.8)	89 (2.2)	54 (1.3)	11 (0.3)	30 (0.7)	29 (0.7)	460 (11.1)
Depression (n=3577)	951 (29.8)	598 (18.7)	518 (16.2)	419 (13.1)	402 (12.6)	238 (7.4)	184 (5.8)	75 (2.4)	37 (1.2)	9 (0.3)	25 (0.8)	25 (0.8)	358 (11.2)
Bipolar Depression (n=506)	135 (30.7)	78 (17.7)	65 (14.8)	71 (16.1)	60 (13.6)	28 (6.4)	31 (7.1)	8 (1.8)	7 (1.6)	1 (0.2)	2 (0.5)	2 (0.5)	49 (11.1)
Mania (n=83)	15 (20.6)	5 (6.9)	9 (12.3)	-12.3	4 (5.5)	5 (6.9)	2 (2.7)	1 (1.4)	3 (4.1)	0 (0)	0 (0)	1 (1.4)	12 (16.4)
Schizophrenia (n=204)	38 (19.9)	16 (8.4)	22 (11.5)	17 (8.9)	18 (9.4)	19 (10.0)	10 (5.2)	3 (1.6)	1 (0.5)	0 (0)	2 (1.1)	0 (0)	22 (11.5)
Schizoaffective Disorder (n=97)	18 (21.2)	5 (5.9)	6 (7.1)	7 (8.2)	6 (7.1)	4 (4.7)	1 (1.2)	0 (0)	4 (4.7)	0 (0)	0 (0)	0 (0)	5 (5.9)
Mixed Affective Disorder (n=38)	7 (20.0)	6 (17.1)	4 (11.4)	7(20.0)	1 (2.9)	3 (8.6)	4 (11.4)	0 (0)	0 (0)	0 (0)	0 (0)	1 (2.9)	3 (8.6)
Post-Partum Disorders (n=27)	7 (30.4)	6 (26.1)	3 (13.0)	3 (13.0)	4 (17.4)	0 (0)	4 (17.4)	0 (0)	0 (0)	0 (0)	1 (4.4)	0 (0)	1 (4.4)
Other (n=101)	26 (29.2)	18 (20.2)	7 (7.9)	11 (12.4)	8 (9.0)	8 (9.0)	5 (5.6)	2 (2.3)	2 (2.3)	1 (1.1)	0 (0)	0 (0)	10 (11.2)

Where: Patients with <=12 treatments included
